# Health and social care coordination for severe and persistent mental illness in Australia: a mixed methods evaluation of experiences with the Partners in Recovery Program

**DOI:** 10.1186/s13033-018-0194-2

**Published:** 2018-04-03

**Authors:** Michelle Banfield, Owen Forbes

**Affiliations:** Centre for Mental Health Research, Research School of Population Health, The Australian National University, Canberra, Australia

**Keywords:** Care coordination, Integration of care, Mental health, Health services, Partners in Recovery, Continuity of care

## Abstract

**Background:**

Care coordination has been identified as a person-centred response to the difficulty in meeting the needs of people with severe and persistent mental illness and complex needs. This study evaluated the processes and outcomes of the Partners in Recovery initiative in the Australian Capital Territory, a program established to improve coordination of health and social care for this population.

**Methods:**

Client, carer and service provider experiences were investigated using a combination of quantitative and qualitative methods. Quantitative data were collected through questionnaires completed by clients (*n* = 25) and service providers (*n *= 14). Qualitative data comprised open-ended written feedback from the surveys, together with semi-structured interviews with selected clients (*n *= 6), carers (*n* = 2), and service providers (*n* = 4). In both study elements, questions focused on dimensions of experience such as communication, continuity and coordination, teamwork and sustainability. Descriptive statistics were calculated for quantitative data; qualitative data were analysed using content analysis.

**Results:**

Clients were satisfied with the program across the majority of experience dimensions, and there was evidence of improved access to coordinated care. Support Facilitators (care coordinators) were central to client and carer reports of the impacts of the program, and to coordination between services through connections built at the individual level. Challenges included difficulties with information continuity, a lack of role clarity for service providers, and uncertainty about the legacy of the program given the absence of formal agreements connecting different services.

**Conclusions:**

The Support Facilitator role was critical to the success of the program. Support Facilitators acted as a source of stability and relational continuity for clients, while also enabling connections with external services through the development of individual level partnerships and personal networks. Systems level coordination was limited by communication difficulties and a lack of formalised infrastructure to support cooperation between services, calling into question the lasting impact of the program for system change.

**Electronic supplementary material:**

The online version of this article (10.1186/s13033-018-0194-2) contains supplementary material, which is available to authorized users.

## Background

Providing services to people with severe and persistent mental illness is complex as it requires both coordinated and collaborative efforts between multiple sectors. This includes primary mental health and physical health care, as well as income support services, employment, education, housing support and non-government sector organisations such as alcohol and drug treatment services [1]. Due to the difficulties in navigating services, care for people with complex needs is often inefficient and lacking. Care coordination has been identified as a person-centred response to this difficulty in meeting the needs of people with severe and persistent mental illness [1].

Coordination in mental health care usually includes flexible care plans that facilitate team services across health and social care boundaries over time [2, 3], and support drawn from several sources, such as family, community, peers and various service providers, is recognised as having the ability to improve a person’s health and well-being outcomes [4].

However, the evidence for the effectiveness of care coordination is currently limited. Programs that have demonstrated success suggest that it is a caring and personal relationship with the care coordinator that is the defining element [4, 5]. The care coordinator is a single, trusted person who helps the client to navigate the system, acting like an anchor to assist the client to better manage transitions between clinicians and services [6]. Recent international reviews concluded that clients derived a sense of security and trust when they were told what to expect and were provided with information to support an active role in their self-management [6], and benefited from support when crossing care boundaries [7]. These studies suggest that both a personal relationship providing regular and continuous care, and encouragement of self-care management may underlie the success experienced via the care coordination model. Research on mental health care coordination programs that specifically target these mechanisms is needed to understand the processes (including barriers and facilitators to implementation) and the outcomes for clients and services.

### Partners in Recovery Program

The Partners in Recovery Program, funded by the Australian Government in the 2011/12 Budget [8], is a mental health care coordination program created to provide tailored, wrap-around care to people with severe and persistent mental illness and complex care needs that had not been adequately addressed. The program was designed to integrate community health and human services, and comprised a consortium of local organisations and service providers within local regions, coordinated by a “Lead Agency” such as a primary health network (regional organisation contracted by Government to plan and coordinate primary health care) [1, 8]. The aim was to coordinate care across the relevant sectors, addressing service delivery gaps. Core components were (1) facilitation of coordinated clinical and support services to deliver ‘wrap-around’ care tailored to the individual, (2) the creation of stronger partnerships and links between these services, (3) improvement of referral pathways to and between services; and (4) the promotion of a community-based recovery model to underpin the services delivered [1]. The initiative required flexible roll-out in individual locations depending on existing services, a client-focused and recovery-oriented model of care, and complementing and coordinating the existing services at each PIR location [1].

At the core of the program’s design was the Support Facilitator (care coordinator), whose primary responsibilities were to assess client needs, manage referrals and develop partnerships for participants’ recovery goals [9]. A client entering the PIR program underwent a detailed assessment of their specific needs such as access to stable housing; the Support Facilitator then located and facilitated access to services to ensure that people with mental illness were not lost to gaps between services. PIR also provided a small amount of ‘flexible funding’, which could be accessed as required for participants to access services and additional supports that could not be sought elsewhere [1]. A PIR evaluation by Brophy et al. [4] reported that the success of PIR depended on ‘having care coordinators who are well prepared for the role, can demonstrate competent practice and achieve better systemic responses focused on the needs of the client, thus addressing the barriers to effective care and treatment across complex service delivery systems’ (p396). A recent study on the nature of the Support Facilitator role concluded that those in the role were creating it according to the nature of local service availability and client needs, but this ad hoc approach was both a strength and a barrier to effective recognition of the role’s importance [9].

The flexible development and implementation of the program according to local systems necessitated evaluation of the processes and outcomes of the PIR care coordination model in individual locations in addition to the National evaluation [1]. This paper reports system level outcomes from an evaluation of the PIR Program in the Canberra region, including client, carer and service provider experiences of improved partnerships between services, implementation of a local model of coordination and improvements in coordinated care.

The key evaluation questions of interest were:

1. Are clients and carers satisfied with the Program?
2. Do clients experience better coordination of their care?
3. Do service providers believe coordination has improved?
4. Are service providers satisfied with the local Partners in Recovery model?
5. What are the barriers and facilitators for effective partnerships and referrals?
6. What are the main factors that may affect sustainability of the model, both within and beyond PIR Program funding?

## Methods

The ethical aspects of the study were approved by The Australian National University Human Research Ethics Committee (2015/148). Written informed consent was obtained from all participants included in the study.

Client, carer and service provider experiences of PIR were investigated using a combination of quantitative and qualitative methods. Quantitative measures comprised client and service provider questionnaires as described below; qualitative data collection consisted of open-ended survey responses, and individual semi-structured interviews with selected clients, carers and service providers.

### Participants and recruitment

Participants in the evaluation comprised three key groups: PIR clients, carers of PIR clients and service providers in the region connected with the PIR Program. The Support Facilitators were located in six community mental health service providers, and referred clients to a wide range of health and social care services throughout the region.

#### Clients

To be eligible for the PIR Program, potential clients had to satisfy five criteria: (1) a severe and persistent mental illness, (2) complex needs requiring substantial services from multiple agencies, (3) recent engagement with services, (4) experienced a failure of coordination between services previously that would be likely to be addressed by PIR, and (5) expressed a willingness to be referred for ongoing treatment and consented to participation [1]. PIR guidelines allowed for a range of indicators of illness severity, persistence and complexity to be used in initial assessment of eligibility for the program, including: diagnosis of a psychotic or other mental illness associated with significant impairment of functioning and that has lasted or is likely to last a number of years; repeated hospitalisations for mental illness within the past 3 years; and/or receipt of a disability pension for mental illness. Clients assessed as eligible received a comprehensive Needs Assessment, including referral for formal diagnosis where necessary [1].

Current and past clients (*N *= 194) in the PIR Program were eligible to participate in the evaluation. Current PIR clients were informed of the evaluation by their Support Facilitators. Invitations to complete the endpoint survey were sent in April 2015 and March 2016 to all clients who had already exited the program.

Participation was initially low due to the requirement for clients to contact the research team to take part in the evaluation (eight participants in the first 3 months of the evaluation, April–June 2015). An amendment to consent procedures that allowed Support Facilitators to pass clients’ contact details to the research team with written consent improved recruitment in the second recruitment round, conducted from January–June 2016.

#### Carers

Carers were recruited through PIR clients. When clients consented to participate in the evaluation, they were asked if they had a carer who may also be interested in participating and if so, were provided with an information sheet and consent form to pass to their carer. Carers participated in interviews separately from PIR clients.

#### Service providers

Service providers were recruited via an advertisement distributed by the PIR Lead Agency to all organisations in contact with the program. This included community organisations employing Support Facilitators and directly providing services to clients, together with broader health and social care organisations to which clients were referred for additional services. The advertisement included a link to an anonymous online survey: to protect service provider confidentiality and encourage honest answers, no demographic data were collected. Participant relationship with the program was assessed using a question that asked for their familiarity with PIR (responses ranged from having a central role to no familiarity).

### Quantitative data collection

Quantitative data were collected using paper and online questionnaires. Client participants could either complete the survey in person with assistance from a researcher, or to be mailed the questionnaire and return using a postage-paid envelope. Service providers completed their survey online.

#### Quantitative measures

The measurement of client experience is one way to assess the quality of care received [10], and satisfaction with care has been found to be positively correlated with how well staff have adhered to treatment guidelines [11]. Key questions 1 and 2 (client satisfaction with care and experience of coordination) were measured using 21 items from the comprehensive survey developed from Wong and Haggerty’s work to identify the dimensions of patient experience, the Canadian Institutes of Health Information (CIHI) Measuring Patient Experiences in Primary Health Care Survey [12]. This survey was chosen over other patient experience measures as it has been found to have the most comprehensive coverage of patient experience, was developed with extensive client, carer and service provider input and was designed for use of specific questions and dimensions of experience most relevant to individual studies [13]. The current evaluation included items from four of the six dimensions of patient experience (interpersonal communication, continuity and coordination, comprehensiveness of services and impacts of care). Participants rated their experience on three-, four- or five-point Likert-type scales.

Client experience data were collected at two time points: a survey at the entry to the evaluation, which for all but one participant was when they had already been in the program for several months, and an endpoint survey upon program exit or conclusion of the evaluation, whichever came first. PIR was designed to be flexible according to client needs and had no prescribed duration, and recruitment was conducted on a rolling basis, therefore the interval between surveys varied. Participants in the first recruitment cohort (2015) had an average of approximately 12 months between midpoint and endpoint surveys. Participants in the second cohort (2016) had 2–4 months between the first and second surveys. Program funding was extended beyond the evaluation period, so not all clients exited prior to the completion of data collection.

The service provider measure of experience consisted of a 14-item self-report questionnaire, comprising questions on coordination and team functioning drawn from the CIHI Attributes of Primary Health Care Provider survey [14], along with three open-ended questions to explore barriers and facilitators for program effectiveness and sustainability. The majority of response options were five-point Likert-type scales or yes/no.

#### Quantitative analyses

Descriptive statistics were calculated in SPSS (v24) for all quantitative measures. Inspection of distributions revealed a substantial negative skew on most items. To address this and provide better consistency for interpretation, all items were dichotomised to create ratings of positive/negative experience. The split of Likert-type scales was guided by item and response wording: most items included yes/no or good/poor descriptors in the response options. For example, a five-point rating scale from “very poor” to “very good” became “poor” (very poor, poor, fair) and “good” (good, very good”) and a three-point scale consisting of “often”, “sometimes” and “never” became “yes” (often, sometimes) and “no” (never). Results reported are the percentage of valid positive responses at each time point. A total of 19 clients completed midpoint surveys and 15 completed endpoint surveys; however, less than a third completed both, therefore data were treated as cross-sectional. Service provider data were collected throughout the evaluation (once per participant).

### Qualitative data collection

Interviews with all participants were conducted by two researchers trained to complete the interviews consistently according to the protocols. Interviews were digitally audio-recorded with participant permission, for later transcription and analysis.

Semi-structured interview protocols were developed for clients, carers and service providers, based on the relevant key evaluation questions relevant. Table 1 presents example questions for each group. Full protocols are provided in Additional file 1.

**Table 1:** Sample questions from client, carer and service provider interviews

Example client and carer questions
1. In your own words, tell me how the program went 2. Was the program what you were expecting? Did you have to change your expectations or goals? 3. What was good about the program? What wasn’t so good? 4. How did it compare to other programs/other support/care you have used in the past?
Example service provider questions
1. Overall, have you felt that the Partners in Recovery Program has been successful? Why? Why not? 2. Do you feel that new/improved partnerships have been forged? What factors have facilitated effective partnerships and referrals? 3. What challenges to successful implementation of the program have you experienced/witnessed? 4. What factors do you think will affect sustainability of the model, both within and beyond PIR Program funding?

#### Qualitative analyses

Qualitative data comprising open-ended survey responses and interview data were analysed using a deductive content analysis approach [15]. A deductive approach was chosen as the primary purpose of the qualitative arm of the study was to further explore the specific questions of interest from the evaluation framework, providing contextual information to support and expand upon the quantitative data. Selected quotes are included where appropriate, identified by participant type (client, carer, service provider) and number within that group.

## Results

### Client and carer satisfaction and experience with coordination

The overall rate of clients agreeing to participate in the study was 17% (32/194). Table 2 presents the demographic characteristics of the participants (*n *= 25) who completed one or more surveys. Seven additional participants who consented to participate in the evaluation did not provide any data and were excluded from the analyses. Participants received services from six different community mental health service providers that were part of the region’s PIR Program. Six of these participants (five female) also completed interviews. Interviewees were referred by Support Facilitators from four of the service providers.

**Table 2:** Demographic characteristics of the sample completing one or more surveys (*n *= 25)

Characteristics	
Age (*M, SD*)	42.82 (12.51)
Gender *(n, %)*	
Male	7 (28)
Female	15 (60)
Not stated/unknown	3 (12)
Aboriginal/Torres Strait Islander (*n, %)*	
Yes	2 (8)
No	16 (64)
Not stated/unknown	7 (28)
Marital status *(n, %)*	
Single	2 (8)
Never married	5 (20)
Separated	1 (4)
Widowed	1 (4)
Not stated/unknown	16 (64)
Participants at each service provider (*n, %)*	
Service Provider 1	4 (16)
Service Provider 2	5 (20)
Service Provider 3	4 (16)
Service Provider 4	2 (8)
Service Provider 5	3 (12)
Service Provider 6	7 (28)

Two carers agreed to participate in interviews at the conclusion of the evaluation: both were female and cared for clients with Support Facilitators from two different services. No other demographic information was collected to maintain confidentiality.

Table 3 summarises quantitative results for clients. Client and carer satisfaction with the program and experience of coordination (key questions 1 and 2) are then described according to the dimensions of experience.

**Table 3:** Client and carer ratings of experience

Dimension and item	Midpoint percentage positive (n), n = 19	Endpoint percentage positive, (n), n = 15
Interpersonal communication		
Time given by services	74% (14)	87% (13)
How services listened	90% (17)	73% (11)
Involvement in decisions about care^a^	83% (15)	80% (12)
Finding out about concerns	90% (17)	87% (13)
Your say in what was important	95% (18)	93% (14)
Taking your concerns seriously	95% (18)	87% (13)
Concerned about your feelings^a^	89% (16)	87% (13)
Discussing goals or priorities	100% (19)	93% (14)
Working out a recovery plan together with Support Facilitator^b^	90% (17)	100% (14)
Continuity and coordination		
Knowing recent history^c^	71% (12)	73% (11)
Known changes in recovery plan recommended by others^c^	77% (13)	93% (14)
Not had to repeat information^a^	89% (16)	73% (11)
Not told different things that didn’t make sense^a^	89% (16)	80% (12)
Services working well together^a^	78% (14)	87% (13)
Services knowing who should do what for your care^a^	72% (13)	87% (13)
Comfort talking about personal problems in services arranged by PIR^a^	83% (15)	80% (12)
Comprehensiveness of services		
Has PIR provided everything you expected	95% (18)	93% (14)
Have you had enough support from services	84% (16)	87% (13)
Impacts of care		
Sense of control	90% (17)	93% (14)
Feeling your recovery plan would make a difference^c^	88% (15)	80% (12)
Confidence in your ability to take care of yourself	95% (18)	80% (12)

^a^One midpoint response missing on this item. ^b^ One endpoint response missing on this item. ^c^ Two midpoint responses missing on this item

#### Interpersonal communication

Interpersonal communication was assessed using nine items that investigated participants’ experience of being listened to, involvement in decision-making and collaboration on plans. Participants were very satisfied with items in this dimension, with more than 80% of participants rating items positively for at least one time point. Collaborative goal-setting and recovery planning received 100% positive responses at mid- and endpoint respectively. Together with the high ratings on having a say on what was important, this suggests that participants felt they were a central part of the recovery-oriented care in the program.

This was supported by comments from interview participants both during and after the program. Clients were positive about their rapport with their Support Facilitators, who they described as “…always looking for ways to improve,” challenging them about things that were not healthy but also listening to their stories and ideas.

*It’s taken time to build trust and accept the help that was offered *+* pro*-*actively use PIR. At first the care plan just seemed like another piece of paperwork, but now I have overcome the shame related to mental health and can use the services effectively.* Client survey participant

*I think [Support Facilitator] and I have worked really well together… it’s definitely evolved and I feel like I’ve always been able to negotiate with her what I’ve wanted, what I haven’t wanted.* Client 5 (endpoint)

However, there was some evidence that rapport was dependent on the success of individual connections. One carer, who described experiences with a range of programs and multiple Support Facilitators, felt that one Support Facilitator did not have a good understanding of how to relate to people with psychotic illness.

*I don’t think he really understood … with people like my son, they take a long time to trust people and a long time to work with because of the nature of the illness. And it takes a long time to get the trust and to actually do something. But they need that contact. And I don’t know if he really understood the whole situation*. Carer 1

#### Continuity and coordination

Client experience of continuity and coordination of care was assessed by seven items that explored information continuity and perceptions of coherent care. Although the majority of ratings were still positive, percentage of positive responses were comparatively lower than for other dimensions: all items scored 80% or fewer positive ratings for at least one time point. In particular, more than a quarter of clients indicated that their history was not available at all providers and they had to repeat information.

*With a new service or worker it’s like starting again. No history was shared*-*or if it was they haven’t read it.* Client survey participant

*I think there needs to be a little bit more communication between the actual services and PIR about how things are working…* Client 6 (midpoint)

By the end of the evaluation, participants reported improvements in the coherence of the care services provided. For client interview participants, this coherence gave a sense of holistic care, focused on them as a person rather than a mental health problem.

*Most programs or services that I’ve accessed have been very specific it’s either been counselling and this is what we do, whereas this, it’s not really counselling. I guess its facilitation and empowerment and reconnection. It’s so holistic that I’ve never experienced anything like it…* Client 5 (midpoint)

Consistent with her description of a poor fit between her son and his Support Facilitator, one carer did not believe that PIR had delivered on its role in care coordination.

*… when we first heard that they were doing the coordinating, because it’s the carer that usually ends up doing all the coordinating…And that’s with everything, it’s not just the health part, it’s the housing, the [social security], whatever…specialists. You’re the one that has to run around and try and coordinate everything and link it all up, and that’s kind of what I thought PIR would maybe have taken that role. But it hasn’t worked out like that.* Carer 1 (endpoint)

#### Comprehensiveness of services

Two items explored clients’ satisfaction with the support and services provided. The overwhelming majority (over 90%) reported that PIR had provided everything they expected and a slightly smaller majority (84–87%) also indicated that the program provided sufficient support.

Most interview participants reported that their Support Facilitators were proactive in their support, making time to find the right services, and demonstrating a “modern” understanding of mental illness that was not just about medication or telling clients what to do without consideration for other influencing factors or for shared decision-making.

*Partners in Recovery is like an advocate. They are, they can speak for you, you tell them what you want and they know exactly where to go, what to do to get it done.* Client 2 (midpoint)

*I’ve always felt like Partners in Recovery really understood mental illness. Really had a very modern understanding of it, that it’s not about somebody coming in telling you what to do, “Take your pills and do this and do that”. It’s like “OK, you’re struggling with that, what can we do to, you know, change your life, tweak it in ways that you can do that better.”* Client 5 (endpoint)

However, one client was equivocal about the benefits of the program, particularly over time, commenting at the endpoint that the program had been better than others, but had not really been very good at referrals or contacts.

*…it went OK. I meet with my support worker usually every 2* *weeks. They’ve arranged some things for me like collecting food packages and some other things…[but] I feel like I’ve been meeting with my support worker less as time goes on and sometimes it’s not often enough because like if I run out of food I don’t really feel like I can contact them like “Hey can you take me out to that place to get a food package” or whatever.* Client 4 (endpoint)

#### Impacts of care

The final three quantitative client experience items assessed how well PIR enabled clients to feel in control of their own health. At least 80% of clients reported positive impacts of the program at both time points. A major theme in interviews was the destigmatising nature of the program, which helped people with their sense of self-efficacy, and one carer also credited the program with a complete turnaround in their son’s life.

*…one of the good things that came out strongly for me was that it was trying to promote me doing something for myself if that makes sense… So rather than something being handed to me on a platter… I had to contribute to it.* Client 1 (midpoint)

*On every level. … he’s caring about his appearance. He’s cooking for himself. The gardening thing. Yeah on every level. I can’t think of one level where it’s not this 125% improvement. Yeah, it’s been amazing.* Carer 2 (endpoint)

Notably, although there was a greater percentage of positive responses for sense of control at endpoint compared with midpoint, feeling that the recovery plan would make a difference and confidence in self-care both decreased from midpoint to endpoint. Comments from interview participants suggest that some clients felt a lack of self-confidence from past experience of the course of mental illness. This may have been exacerbated by funding uncertainty for the program, giving clients concern that there would no longer be support available if they needed it, undermining the self-efficacy built by the program.

*… you know everything’s till the end of June because we don’t know what’s happening… And that… and we know as a consumer, oh those of us that are able to understand, that all this stuff’s uncertain. But that’s how we… we live and feel the way our… the way the organisations that help us feel, because they live with uncertainty all the time about their funding…* Client 3 (midpoint)

### Service provider satisfaction and experience with coordination

Fourteen service providers completed the survey; all were at least “somewhat familiar” with the program and five indicated that it was central to their role, with a strong working knowledge. In addition, four service providers completed in-depth interviews at the conclusion of the evaluation.

Table 4 presents quantitative results for service providers (key questions 3 and 4), subdivided into the dimensions of service provider experience; these are supported by quotes from interviews.

**Table 4:** Service provider ratings of experience

Dimension and item	Number of responses	Percent positive responses (n)
Team functioning		
Satisfaction with		
How program members communicate	10	90% (9)
Others’ understanding of scope of practice	12	92% (11)
Understanding of own role in team	11	73% (8)
Understanding of others’ role in team	10	90% (9)
Frequency of team meetings	10	80% (8)
Collaboration in setting goals	10	60% (6)
Participation in administrative decision-making	10	50% (5)
Service delivery coordination		
Extent able to coordinate for planning and providing care	11	91% (10)
Awareness of health and social care consultations	10	40% (4)
Communication with external providers	11	82% (9)
Availability of same information between providers	12	58% (7)
Collaboration with external providers to set goals and recovery plans	12	75% (9)
Records available	8	75% (6)
No problems due to poor coordination	8	100% (8)

#### Team functioning

Service provider satisfaction with team functioning was measured by seven items that explored communication, role perceptions and collaborative decision-making. The majority of participants were satisfied with team functioning in the PIR Program and some interview participants felt that the consortium model was successful.

*I actually enjoy the model, and one of the successes I think is, one of the good things that’s challenging and it’s great as well, is having a whole lot of different organisations involved, because we all come together and then we all… so I have access into all these other organisations through these other workers that I know very well, get to know very well.* Service provider 3

However, two areas scored substantially lower positive responses: collaboration on goals and involvement in administrative decision-making. This was reflected in comments made by interview participants, who were concerned that significant turnover of staff at the Lead Agency created inefficiencies in program administration and a lack of clear leadership.

*And as each change of worker in the agency, they’ve required us to do things, and then when they’ve left the requirement to do that thing has not existed anymore.* Service provider 3

*I would like to say it’s been successful from my personal point of view, because I’ve worked quite hard to make it successful. I think the reason I say that is more to do with my hard work than amazing leadership, which I think has been really lacking. So I think that’s compromised the model. I think the model in theory is a positive model, but in practice there have been many limitations that haven’t been resolved… In the absence of leadership and you know capacity to resolve things.* Service provider 2

#### Service delivery coordination

Coordination from the service delivery perspective was measured by seven items investigating information continuity and collaboration on care. As for client experience, service provider responses suggest some issues with information continuity: for example, only 40% of providers said they were aware of 60% or more of their clients’ health and social care consultations and only 58% reported that the same information was available to all providers. Comments from interview participants suggested that this may have stemmed, at least in part, from challenges in the program management software.

*… I have found that communication is usually one*-*sided. I am not kept informed as to information which may affect my ability to make progress with a client. We also receive very little to no feedback through the program as to the impact on the client and whether we can improve the way we work with them.* Service provider survey respondent

*The data program that we work to was designed not specifically for PIR, but it was designed more broadly for a range of programs within [the Lead Agency]. So there’s been lots of tweaking and so on… We need to be able to put the information in the right places on the database, and sometimes it doesn’t happen, so that creates more work and more downtime for …staff to go through and find the information.* Service provider 1

Despite this, three quarters or more of respondents were positive about collaborative care, and there were no reported problems due to poor coordination. As one interviewee put it, the program was very successful at building relationships due to the “personal approach” of the Support Facilitators.

*We’ve learnt a lot of about the services that are out there, and I think also we’ve built up a good level of relationship with the various services. You know we get to know a lot of the service workers around the ACT. And I think you know that’s where part of the success is, too, is connecting with other services and taking a more personal approach, and just wading through all the mystical stuff, or the stuff that people just can’t deal with.* Service provider 1

### Barriers and facilitators to effective partnerships and referrals

As described above, the PIR consortium model was thought to facilitate partnerships, creating connections between organisations. However, some service providers thought the model was not without its challenges, including a lack of clarity of the precise role of the Support Facilitator and a lack of services available for coordination.

*I think first you have to be sure that there are all those services to coordinate in the first place…Do all the services exist? … If you want to coordinate something there has to be something there to coordinate, I guess… For the model to work it has to be available.* Service provider 4

One aspect of the program that was mentioned as a particular facilitator was the “flexible funds” (brokerage) available to help clients with immediate needs. Service providers noted that this was unusual and facilitated the referral process.

*I think in large part that’s been underpinned by the use of brokerage, because for the first time … Partners in Recovery was the first time that I’ve ever heard of a program where we had an appreciable amount of money allocated for each person that we work with to assist. So what that helped with in terms of local networks and partnerships with other orgs and other services is we were able to assist.* Service provider 2

Most service providers reported positive experiences connecting with the various government and community services associated with the PIR Program, but some did feel that their lack of role clarity and a lack of formalised agreements hindered their relationships with clinical teams.

*…the challenge of earning our stripes and getting involved with the mental health team… So it was the mismatch thing, the classic NGOs versus clinical teams. I think we just, we had all this responsibility but no real authority like who were we, who was Partners in Recovery?… The challenge was trying to describe the program, I guess, like what is a Support Facilitator.* Service provider 4

*… really haven’t been able to work close enough with [the Lead Agency] to look at developing some solid agreements with people like the hospitals and the health service, and some of the other people, Indigenous organisations and other services around the ACT… sometimes it’s good to have something like a service level agreement or an MOU in place, and particularly with places like the hospital, where they have you know protocols and procedures in place…* Service provider 1

### Sustainability

The primary threat to the coordination model created by the PIR Program was seen to be funding. The program was identified as “in scope” for the National Disability Insurance Scheme (NDIS), a National program to provide flexible packages of care to Australians with disability, including psychosocial disability. An extension of funding for PIR beyond the initial 5 year period was granted only to facilitate transitioning of eligible clients to the NDIS and to other services for those not eligible. Service providers struggled to reconcile themselves to this uncertainty after the program’s perceived success. The program’s lasting effects on the system also seemed unclear, and this was a source of disappointment to the people involved in the program, who felt that it had potential to have a strong legacy.

*We still have a role as a co*-*ordinator of supports under the NDIS, but even that, we don’t know what’s going to happen at the end of next year, the 30th of June, when we definitely won’t be having jobs in PIR anymore …So what happens to the people who we were co*-*ordinating supports for and have had… you know generally we’re doing that for people who we’ve had a long relationship with in PIR, and then we just move into co*-*ordination of supports.* Service provider 3

*Part of the program is systems change and I’m not sure what’s come of that. We had a really good chance to change the system, we had a lot of funding so my concern is*—*and it’s been for a while*—*what is the legacy of Partners in Recovery, what’s going to be left after we’re gone, did we change anything significantly that the whole system could have held onto and gone “Wow, that was really great and Partners in Recovery helped change that.”* Service provider 4

## Discussion

Reflection on the PIR Program offers an opportunity to examine the successes and shortcomings of a local coordination of care model for severe and persistent mental illness, and its broader relevance to coordination of care programs internationally. Evaluation findings reflect a mostly positive overall experience with the PIR Program for clients, carers and service providers. Participants were satisfied with the program across the majority of experience dimensions, and there was evidence of improved access to coordinated care. Support Facilitators were central to client and carer reports of the impacts of the program and a recovery-oriented approach to care. However, service providers (including Support Facilitators) were not confident that the positive changes they had experienced would be sustained beyond the program’s end. This lack of confidence may reinforce arguments elsewhere that “relationship-centred care” is crucial to the success of care coordination initiatives for mental health [9], and that system-level partnerships may not be as effective without the care coordinator role providing an experience of relational continuity to the client.

Given that successful implementation of care coordination is experienced by clients as continuity of care [3], experiences of continuity in different domains can provide insight into the mechanisms underpinning the effectiveness of these programs. The design of the PIR program reflects evidence that the care coordinator relationship (relational continuity) is the defining element of successful coordination models [4, 5, 16]. Consistent with claims that the continuity of contact between care coordinator and client is critical to the success of flexible and holistic delivery of care [3, 6, 7], trusting relationships with Support Facilitators appeared to be a central factor behind client satisfaction with PIR, helping to build a sense of self-efficacy and confidence about their future. The negative experiences reported, which centred on a poor “fit” between client and Support Facilitator, further emphasise the centrality of the care coordinator role in client experience of these models. This suggests that in order to maximise effectiveness, services looking to implement a care coordination model should focus on the workers placed in the care coordinator role, not only in terms of their skills in navigating fragmented systems, but in their ability to foster trust and relate to their clients. As acknowledged by Smith-Merry et al. [9], lived experience of mental health problems (peer work) has a strong part to play in these boundary spanning or brokerage roles, as this provides a means for developing strong connections that are different from clinical relationships. Further, there is a need to monitor the suitability of the care coordinator-client match, particularly when a program is not producing anticipated outcomes, to identify areas of poor interpersonal or skills fit.

Consistent with findings from other studies on care coordination, which report that availability and consistency of information between providers is a key challenge that can undermine the administration of care coordination initiatives [16, 17], some of the lowest ratings for client satisfaction regarded information continuity. More than a quarter of clients indicated dissatisfaction with knowledge of their recent history across services, and a need to repeat information when visiting new providers. Items related to information continuity were also among the poorest rated by service providers, who identified problems with the available information sharing systems. As healthcare systems increasingly rely on electronic information to support multidisciplinary care [e.g., 18], improved management of client information and the utility of the systems used in that management are areas still in need of improvement. The availability and transfer of information is not enough on its own to ensure coordination [16, 19], and clients and providers across a range of studies and settings consistently report frustration about the poor availability and quality of information available [20]. To avoid perpetuating the problem they are created to address, it is thus important that care coordination programs specifically address the information management practices, including for example client record software and protocols for the sharing of client histories and needs assessments. Such design should account for local contextual influences and be responsive to the needs of clients, families and providers, as evidence suggests that top-down, standardised protocols are rarely effective or acceptable [16, 21].

Building self-management skills and overcoming stigmatising beliefs about their own mental health were key topics raised by clients in open-ended feedback. Clients described the sense of “empowerment and reconnection” gained through working with PIR, focused particularly on Support Facilitators’ assistance in navigating and building bridges across the fragmented mental health care system. This is consistent with the program’s person-centred approach. However, this contrasts with the findings from interviews with Support Facilitators in Western Sydney: in this study, the authors noted a lack of explicit reference to recovery orientation by Support Facilitators and expressed concern that recovery may not be receiving adequate promotion within the program [9]. Evidence from the current study and the Victorian programs [17] suggests that most clients do experience recovery during the program, and recognise the areas in which having a “partner”, as suggested by the program title, is of most benefit. Crossing care boundaries and accessing new services is often a source of significant stress and uncertainty for clients [8]. As described above, it is therefore crucial to ensure that programs seeking to take a recovery-oriented approach to coordination embed workers with whom clients can relate.

A key aim for the PIR Program was to develop partnerships and linkages between services to effect system change [1]. This system reorientation is described in the Victorian PIR White Paper as an “enduring challenge” requiring sustained effort [17]. Overall, service providers reported that coordination of care was successful in the Canberra region PIR Program due to the dedication and hard work of Support Facilitators, but lasting service level connections were not developed as effectively. The consortium model had facilitated the growth of close relationships between PIR and staff in external services, but the Support Facilitator role was critical for creating connections and relationships to enable cooperation across services, and consistent with findings from Western Sydney PIR, Support Facilitators experienced difficulties with their lack of role definition [9]. Formal infrastructure and agreements supporting service-level cooperation were intended to primarily be the responsibility of the Lead Agency [1], but service providers identified some issues at the Lead Agency level, reporting that it was Support Facilitators who were central in building partnerships for coordinating care. This resonates with Brophy et al. argument for the need for a dedicated “boundary spanning” role in care coordination projects [4], responsible for building formal agreements and supporting infrastructure to embed lasting connections and collaboration between mental health service providers. The same conclusion has also been drawn in primary health care studies for complex chronic illness more broadly [6, 7, 16].

These strengths and challenges have important implications for the sustainability and lasting impact of PIR on care coordination programs broadly. Kania and Kramer argue that for any large coordination of care initiative, “the expectation that collaboration can occur without a supporting infrastructure is one of the most frequent reasons why it fails” [22]. As observed in other studies on the PIR program, it was implemented with little system preparation or workforce planning, and many of the issues such as poor role definition and acceptance, and inadequate information management processes reflect the haste with which it was developed and rolled out [9]. Findings from this and other studies on the PIR model support the need for careful planning of protocols, work roles and information management, developed collaboratively with clients, families and services providers and designed with sufficient flexibility to address the local context. Individual relationships between client and coordinators, and between actors in the system are always likely to influence the success of coordination, leaving the care coordination programs vulnerable to failure when individuals leave, but improved planning and supporting infrastructure may help to reduce the effect.

### Limitations

The strength of these findings is limited by the small number of participants recruited, although the participation rate was similar to contemporary studies conducted with people with serious mental illness [23] and health professionals [24]. The small numbers necessitated collapsing of Likert-type scales to dichotomous variables, but this resulted in a loss of variability and a less nuanced understanding of response trends. In addition, lower than expected participation in the interviews, particularly over multiple time points, restricted the scope of qualitative analyses. Analyses were confined to deductive examination of data for contextual information on the evaluation dimensions rather than an inductive thematic analysis, which may have given a richer understanding of experience.

A potential bias may also have been introduced by the Support Facilitators’ selection of client participants. Clients entering the program were often experiencing acute episodes of mental illness and were judged to be “too unwell” to be referred to the evaluation by the Support Facilitators. Whilst triaging helped ensure the safety of participants and reduce the burden of questionnaires they had to complete, this limited the amount of data collected across the entire evaluation and substantially reduced opportunities to assess changes in outcomes. Future work with care coordinators should consider embedding evaluation processes within service contacts to reduce the sampling effect and allow people who wish to participate the opportunity to do so with appropriate support.

The involvement of the Lead Agency in advertising the service provider participation opportunities may also have influenced who responded to the invitation, and the nature of their feedback. However, there were both positive and negative findings, and participants were willing to comment on aspects of the program which they felt did not work as well, providing balance.

## Conclusions

Evidence from clients, carers and service providers suggests that the PIR care coordination model is successful in meeting its aim to provide tailored, wrap-around care to people experiencing severe and persistent mental illness, but the success is driven by the Support Facilitators. Support Facilitators developed strong, respectful relationships with most clients and carers, and partnerships with a broad range of health and social care services. Coordination was thus underpinned by strong relational continuity and management continuity, but this was weakened by poor communication (information continuity), and there was doubt that the system changes would endure beyond the program funding.

This has important implications for care coordination programs with system-level outcomes amongst their core aims: although many such initiatives focus on the role of information and its ease of transfer in the digital age, success is still dependent on the relationships between clients, families and providers, and assigning responsibility for managing both the information and the relationships. In fragmented health and social care systems where poor communication is a defining feature, in at least the medium term, there will continue to be a need for the care coordinator role to span the boundaries of services and ensure people with complex needs are supported to receive the care they need.

## Additional file

**Additional file 1.** Interview protocols.

## Abbreviations

ACT: Australian Capital Territory
CIHI: Canadian Institutes of Health Information
NDIS: National Disability Insurance Scheme
PIR: Partners in Recovery
SPSS: Statistical Package for the Social Sciences

## References

[1] Australian Government: Partners in Recovery (PIR): Coordinated support and flexible funding for people with severe, persistent mental illness and complex needs initiative. Program guidelines for the engagement of PIR Organisations 2012–2013 to 2015–2016. Canberra: Department of Health and Ageing; 2011.

[2] Banfield MA, Gardner KL, Yen LE, McRae IS, Gillespie JA, Wells RW (2012). Coordination of care in Australian mental health policy. Aust Health Rev.

[3] Haggerty JL, Reid RJ, Freeman GK, Starfield BH, Adair CE, McKendry R (2003). Continuity of care: a multidisciplinary review. BMJ.

[4] Brophy L, Hodges C, Halloran K, Grigg M, Swift M (2014). Impact of care coordination on Australia’s mental health service delivery system. Aust Health Rev.

[5] Craig C, Eby D, Whittington J (2011). Care coordination model: better care at lower cost for people with multiple health and social needs.

[6] Haggerty JL, Roberge D, Freeman GK, Beaulieu C (2013). Experienced continuity of care when patients see multiple clinicians: a qualitative metasummary. Ann Fam Med.

[7] Haggerty JL (2012). Ordering the chaos for patients with multimorbidity. BMJ.

[8] Australian Government (2011). Budget 2011–2012: delivering better hospitals, mental health and health services.

[9] Smith-Merry J, Gillespie J, Hancock N, Yen I (2015). Doing mental health care integration: a qualitative study of a new work role. Int J Mental Health Syst.

[10] Manary MP, Boulding W, Staelin R, Glickman SW (2013). The patient experience and health outcomes. N Engl J Med.

[11] Jha AK, Orav EJ, Zheng J, Epstein AM (2008). Patients’ perception of hospital care in the United States. N Engl J Med.

[12] Wong ST, Haggerty J (2013). Measuring patient experiences in primary health care: a review and classification of items and scales used in publicly-available questionnaires.

[13] Gardner K, Parkinson A, Banfield M, Sargent GM, Desborough J, Hehir KK (2016). Usability of patient experience surveys in Australian primary health care: a scoping review. Aust J Prim Health.

[14] Johnston S, Burge F (2013). Measuring provider experiences in primary health care: report on the development of a PHC provider survey for the Canadian Institute for Health Information.

[15] Liamputtong P, Ezzy D (2005). Qualitative research methods.

[16] Banfield M, Gardner K, McRae I, Gillespie J, Wells R, Yen L (2013). Unlocking information for coordination of care in Australia: a qualitative study of information continuity in four primary health care models. BMC Fam Pract.

[17] Victorian Partners in Recovery Organisations (2016). Larter consulting: a white paper on partners in recovery in Victoria.

[18] Australian Government. Concept of operations: relating to the introduction of a personally controlled electronic health record system. In. Australian Government Department of Health and Ageing, eds. National E-health transition authority Ltd. Canberra: Commonwealth of Australia; 2011.

[19] Reid RJ, Haggerty JL, McKendry R (2002). Defusing the confusion: concepts and measures of continuity in healthcare.

[20] Waibel S, Henao D, Aller MB, Vargas I, Vazquez ML (2012). What do we know about patients’ perceptions of continuity of care? A meta-synthesis of qualitative studies. Int J Qual Health Care.

[21] Greenhalgh T, Hinder S, Stramer K, Bratan T, Russell J (2010). Adoption, non-adoption, and abandonment of a personal electronic health record: case study of HealthSpace. BMJ.

[22] Kania J, Kramer M. Collective impact. In: Stanford social innovation review Winter; 2011.

[23] Thomas N, Foley F, Lindblom K, Lee S (2017). Are people with severe mental illness ready for online interventions? Access and use of the Internet in Australian mental health service users. Australas Psychiatry.

[24] Sahin D, Yaffe MJ, Sussman T, McCusker J (2014). A mixed studies literature review of family physicians’ participation in research. Fam Med.

