# Supplementary material for: Health and social care coordination for severe and persistent mental illness in Australia: a mixed methods evaluation of experiences with the Partners in Recovery Program
Source: Int J Ment Health Syst. 2018 Apr 3;12:13. doi: 10.1186/s13033-018-0194-2 (PMC5883333; doi:10.1186/s13033-018-0194-2)
Supplement: Supplementary file 1 — Additional file 1. Interview protocols. [file 13033_2018_194_MOESM1_ESM.docx]

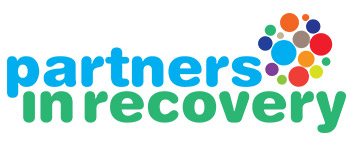


**Evaluation of the ACT**

**Partners in Recovery (PIR)**

**Program**

**Participant Journeys**

**Midpoint Interview**

| **** |  | [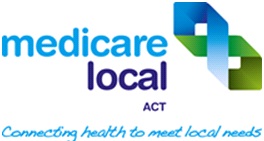](NULL) |
| --- | --- | --- |

# Instructions

**Introduction**:

My name is John, and I work on the Partners in Recovery study at ANU. Before we start, I’ll just remind you that this interview will take about 30 minutes. With your permission, we’ll be recording it, so that we don’t have to rely on my notes and memory. Is this ok?

Remember that our study is about finding out how well the program is running. We want to hear people’s experiences, whether they are good or bad. That way we can suggest any changes that might make the program work better.

We find that it’s better just to let people tell their stories without too much interruption. There are a few questions that I will ask, though. I might also jot down some notes on things you say. Then I can come back and get more information if I need to. Does that sound ok?

Does all of that sound ok, and are you happy to continue?

Alright, you have now been in the program for a little while now. Today’s interview will be about how you feel it has been working for you so far.

1. In your own words, tell me how the program has been going so far:
   1. What was your first impression?
   2. Has your first impression lasted or has it changed?
   3. Has the program been what you were expecting?
   4. Have you had to change your expectations or goals? In what way(s)?
   5. What kinds of referrals/services have you used so far?
   6. What has been good about the program so far?
   7. What hasn’t been so good? What has been difficult?
   8. How has it compared to other programs/other support/care you have received in the past?
   9. Do you have any suggestions for how the program might be improved?

# Thank you!

Thank you – that was the end of your midpoint interview. Do you have any questions about our interview? Do you have any questions about the overall study?

If you think of anything, you can always contact Dr Michelle Banfield. Dr. Banfield’s contact details are on the information sheet provided.

Your Support Facilitator will let us know when you have reached the end of the program. We will contact you then about doing the final survey and interview.

Until then – all the best!


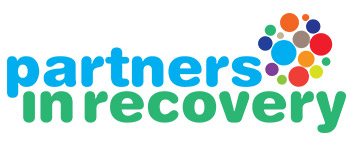


**Evaluation of the ACT**

**Partners in Recovery (PIR)**

**Program**

**Participant Journeys**

**Endpoint Interview**

| **** |  | [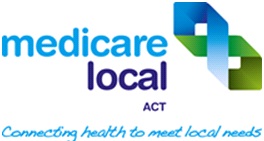](NULL) |
| --- | --- | --- |

# Instructions

**Introduction**:

My name is John, and I work on the Partners in Recovery study at ANU. Before we start, I’ll just remind you that this interview will take about 30 minutes. With your permission, we’ll be recording it, so that we don’t have to rely on my notes and memory. Is this ok?

Remember that our study is about finding out how well the program is running. We want to hear people’s experiences, whether they are good or bad. That way we can suggest any changes that might make the program work better.

We find that it’s better just to let people tell their stories without too much interruption. There are a few questions that I will ask, though. I might also jot down some notes on things you say. Then I can come back and get more information if I need to. Does that sound ok?

Does that all sound ok, and are you happy to continue?

Alright, you have now completed the program. Today’s interview will be about how you feel it went.

1. In your own words, tell me how the program went:
   1. Did it get better as you went along? Worse?
   2. Was the program what you were expecting?
   3. Did you have to change your expectations or goals? In what way(s)?
   4. What kinds of referrals/services did you use?
   5. What was good about the program?
   6. What wasn’t so good? What was difficult?
   7. How did it compare to other programs/other support/care you have used in the past?
   8. When you made suggestions for improvement, did anything change?
   9. Do you have any more suggestions, now that you have finished the program? Do you have anything that you would like to add?

# Thank you!

Thank you – that was the end of your endpoint interview. Do you have any questions about our interview? Do you have any questions about the overall study?

If you think of anything, you can always contact Dr Michelle Banfield. Dr. Banfield’s contact details are on the information sheet provided.

Thank you once again for being a part of our study. Your story is really important for us to be able to work out whether the program is running well. It will also help us to find out what could be improved.

The results of the study will be available on ACACIA’s website. The URL is on the information sheet. They will also be in the Insight Newsletter. The link to this is also on the website.

All the best.


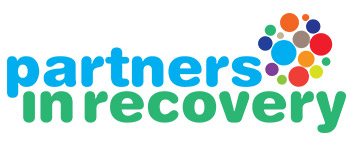


**Evaluation of the ACT**

**Partners in Recovery (PIR)**

**Program**

**Service Provider Interview**

| **** |  | 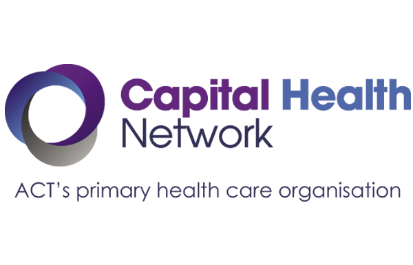 |
| --- | --- | --- |

# Instructions

**Introduction**:

My name is Niah, and I’m the Project Manager for this evaluation of the ACT Partners in Recovery Program. You’ve read the information sheet before you consented, but just to remind you – this interview will take around 30 minutes to complete. We’ll be recording it, so that we can then transcribe what you’ve said and we don’t have to rely on my notes and memory.

We just want to make it clear before we start that our study doesn’t make any assumptions about whether the program is running well or not. There’s no pressure for us to report back positively about the program - it’s far more important to us that we find out the truth of people’s experiences. This goes for whether they are good experiences or bad. That way good recommendations can be made about future funding for the program. We can also make suggested changes to make the program work better once we’ve got a good idea of the strengths and weaknesses.

During this interview, I’ll be asking a handful of questions, but we find that people give better information when we let them just tell their stories without too much interruption, so as much as possible I’ll be letting you talk and recount the story of your experience with the program. I might also jot down some notes on things you say, so that I can come back and get more information if I need to. Does that sound ok?

Alright, if you’re happy to do so, let’s begin.

1. Firstly, what is your involvement with the Partners in Recovery Program? *(Please keep in mind that it is best that you don’t identify the organisation you work for, your specific role within that organisation, or names/details of any other individuals. If you do inadvertently identify these things, they will be removed from any reporting of the interview so as to protect confidentiality).*
2. How would you say that the Partners in Recovery Program has differed from the standard business that your organisation does?
3. Overall, have you felt that the Partners in Recovery Program has been successful? Why? Why not? Are you satisfied with the model? What is the general feeling within your organisation about the model/program?
4. From what you have experienced, what factors have facilitated effective partnerships and referrals?
5. What challenges to successful implementation of the program have you experienced/witnessed?
6. What factors do you think will affect sustainability of the model, both within and beyond PiR Program funding?
7. Do you feel that a local, recovery-based model of coordination has been successfully implemented?
8. What, in your opinion, are the key features of the model of coordination?
9. Does implementation of the coordination model depend on ongoing Program funding?
10. Has coordination of care improved according to the local model?
11. Do you feel that new/ improved partnerships have been forged?
12. Is there anything else that you would like to add?

# Thank you!

That was the end of the ACT Partners In Recovery Evaluation, Service Provider Interview. Do you have any questions about our interview, or about the overall study?

If you think of any questions, you can always contact myself or Dr Michelle Banfield, who is Chief Investigator of the study. Dr. Banfield’s contact details are on the information sheet provided.

Thank you again for taking the time to complete this survey. Your responses will give us valuable insight into the implementation of the PIR Program in the ACT.

All the best!
